# Left atrial myxoma associated with multivessel coronary embolism confirmed by cardiac magnetic resonance imaging: a case of myocardial infarction with non-obstructive coronary arteries

**DOI:** 10.1093/ehjimp/qyae124

**Published:** 2024-12-02

**Authors:** Annalisa Pasquini, Andrea Caffè, Monica Filice, Rosa Lillo, Rocco Antonio Montone, Giovanni Alfonso Chiariello, Natalia Pavone, Marialisa Nesta, Maria Grandinetti, Piergiorgio Bruno, Francesco Burzotta, Massimo Massetti

**Affiliations:** Department of Cardiovascular Medicine, Fondazione Policlinico Universitario A. Gemelli, IRCCS, Largo Agostino Gemelli 8 00168, Rome, Italy; Department of Cardiovascular and Pulmonary Sciences, Catholic University of the Sacred Heart, Rome, Italy; Department of Cardiovascular Medicine, Fondazione Policlinico Universitario A. Gemelli, IRCCS, Largo Agostino Gemelli 8 00168, Rome, Italy; Department of Cardiovascular Medicine, Fondazione Policlinico Universitario A. Gemelli, IRCCS, Largo Agostino Gemelli 8 00168, Rome, Italy; Department of Cardiovascular Medicine, Fondazione Policlinico Universitario A. Gemelli, IRCCS, Largo Agostino Gemelli 8 00168, Rome, Italy; Department of Cardiovascular Medicine, Fondazione Policlinico Universitario A. Gemelli, IRCCS, Largo Agostino Gemelli 8 00168, Rome, Italy; Department of Cardiovascular and Pulmonary Sciences, Catholic University of the Sacred Heart, Rome, Italy; Department of Cardiovascular Medicine, Fondazione Policlinico Universitario A. Gemelli, IRCCS, Largo Agostino Gemelli 8 00168, Rome, Italy; Department of Cardiovascular Medicine, Fondazione Policlinico Universitario A. Gemelli, IRCCS, Largo Agostino Gemelli 8 00168, Rome, Italy; Department of Cardiovascular Medicine, Fondazione Policlinico Universitario A. Gemelli, IRCCS, Largo Agostino Gemelli 8 00168, Rome, Italy; Department of Cardiovascular Medicine, Fondazione Policlinico Universitario A. Gemelli, IRCCS, Largo Agostino Gemelli 8 00168, Rome, Italy; Department of Cardiovascular and Pulmonary Sciences, Catholic University of the Sacred Heart, Rome, Italy; Department of Cardiovascular Medicine, Fondazione Policlinico Universitario A. Gemelli, IRCCS, Largo Agostino Gemelli 8 00168, Rome, Italy; Department of Cardiovascular and Pulmonary Sciences, Catholic University of the Sacred Heart, Rome, Italy; Department of Cardiovascular Medicine, Fondazione Policlinico Universitario A. Gemelli, IRCCS, Largo Agostino Gemelli 8 00168, Rome, Italy; Department of Cardiovascular and Pulmonary Sciences, Catholic University of the Sacred Heart, Rome, Italy

**Keywords:** cardiac myxoma, coronary embolism, myocardial infarction, MINOCA, CMR

## Abstract

**Aims:**

Explore the diagnostic value of multimodal imaging in identifying considerably rare causes of myocardial infarction.

**Methods and results:**

We report a case of myocardial infarction with non-obstructive coronary arteries (MINOCA) probably due to coronary embolism associated with a left atrial myxoma. A 56-year-old male presented with non-ST-elevation myocardial infarction, with coronary angiography showing mild coronary atherosclerosis without significant epicardial stenosis. Transthoracic echocardiography and cardiac magnetic resonance imaging (CMR) revealed a large left atrial mass, suspected to be an atrial myxoma. CMR also showed an ischaemic pattern involving multiple coronary territories, suggesting coronary embolism as the cause of the MINOCA. The patient underwent successful surgical excision of the left atrial mass, and histopathology confirmed the diagnosis of cardiac myxoma.

**Conclusions:**

This case highlights the relevance of CMR in detecting ischaemic patterns in patients with a working diagnosis of MINOCA and underlines the diagnostic value of multimodal imaging in identifying considerably rare causes of myocardial infarction, such as myxoma-associated coronary embolism.

## Case presentation

We present a case of myocardial infarction with non-obstructive coronary arteries (MINOCA) due to a left atrial myxoma-associated coronary embolism, where an embolic ischaemic pattern was confirmed by cardiac magnetic resonance imaging (CMR). This case is notably rare because a suspected coronary embolism from a cardiac myxoma was confirmed by the ischaemic pattern involving multiple coronary territories on CMR.

A 56-year-old male patient was admitted to the emergency department with left-sided stabbing chest pain that was neither aggravated by finger pressure nor relieved by position. He denied recent fever and flu-like symptoms. Blood pressure was 130/80 mmHg, heart rate was 66 bpm, peripheral oxygen saturation was 98% on room air, and body temperature was normal.

The patient had a history of type II diabetes mellitus and systemic arterial hypertension, without any previous cardiovascular events.

The electrocardiogram performed in the emergency department showed sinus rhythm, possible previous lateral necrosis, and non-specific ventricular repolarization abnormalities, while the chest X-ray was within normal limits. High-sensitivity troponin I was 422 and 3415 ng/L (upper limit of normal 57 ng/L) in two consecutive determinations within 1 h. Other routine laboratory tests were within normal limits, except for a mild neutrophilic leukocytosis (white blood cell count 10.8 × 109/L).

**Figure 1:**
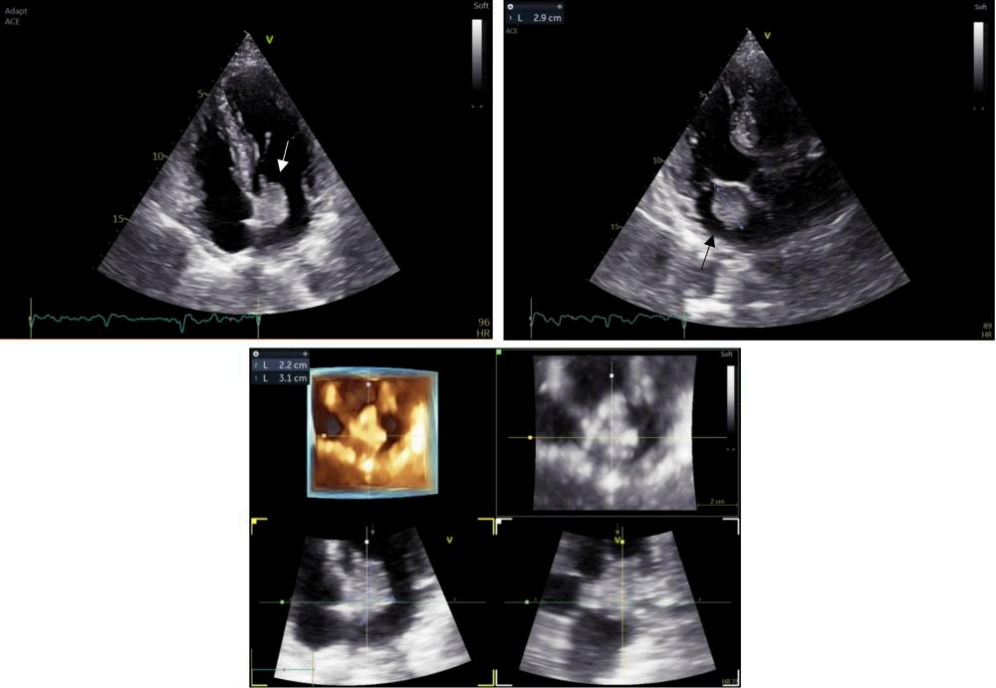
Transthoracic echocardiography. Presence of a left atrial oval formation.

Transthoracic echocardiography (*Figure 1* and *Videos 1 and 2*) showed a voluminous left atrial echogenic mass with a large implantation site on the inferior interatrial septum and a floating and irregular border, in the absence of mitral valve plane involvement or haemodynamic compromise. Left ventricular ejection fraction was preserved without regional wall motion abnormalities. All other findings were within normal limits.

The patient was therefore admitted to the cardiac intensive care unit and underwent coronary angiography the following day, which revealed a left-dominant circulation with mild coronary atherosclerosis in the absence of significant epicardial lesions (*Videos 3 and 4*). High-sensitivity troponin I peaked on Day 1 (18 248 ng/L).

Coronary computed tomography angiography confirmed the absence of significant epicardial atherosclerosis and the presence of a left atrial oval formation (29 × 22 mm) involving the atrioventricular plane, in contact with the posteromedial commissure and the A3 and P3 mitral leaflets.

**Figure 2:**
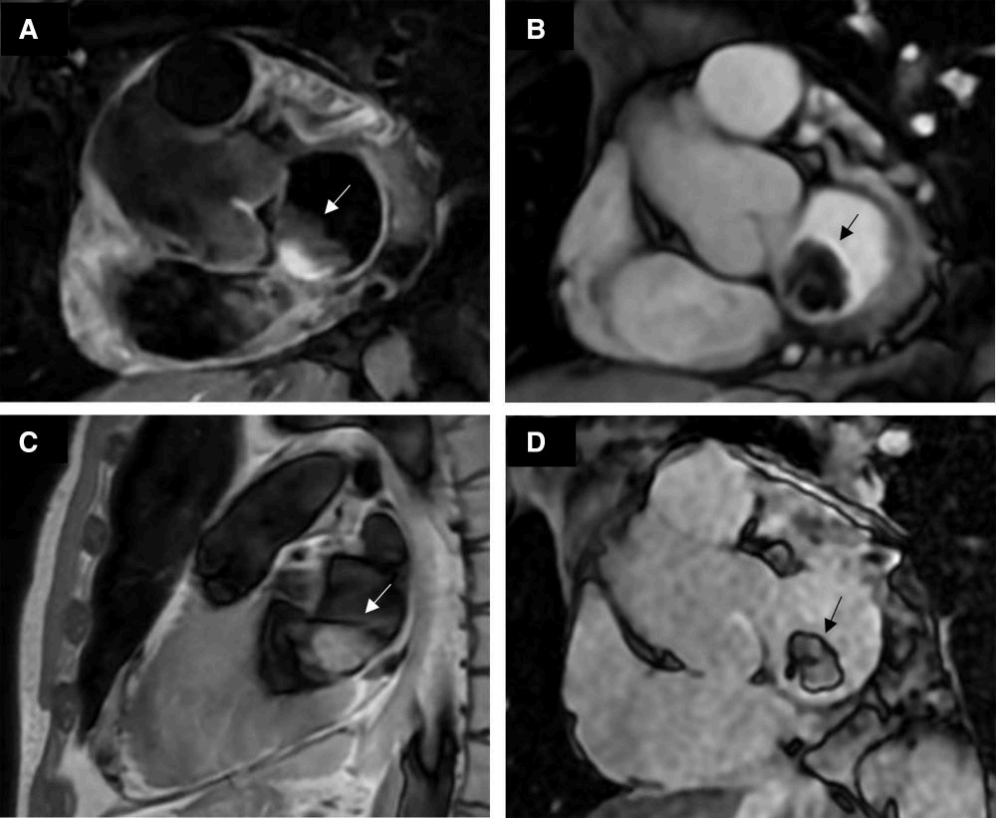
CMR, left atrial mass. CMR T2-weighted black-blood image (*A*), cine SSFP image (*B*), T1-weighted image (*C*), and myocardial delayed enhancement image (*D*) showing the left atrial mass suspected to be a left atrial myxoma.

CMR imaging showed a highly mobile floating solid mass (28 × 20 mm in four-chamber view) with lobulated margins, broadly in contact with the interatrial septum, caudal to the oval fossa and extending to the junction between the anterior mitral leaflet and the valvular annulus, partially exceeding the latter during diastole. The mass was isointense on T1-weighted images and hyperintense on T2-black blood, with mild eccentric enhancement (*Figure 2*). This mass was suspected to be an atrial myxoma.

**Figure 3:**
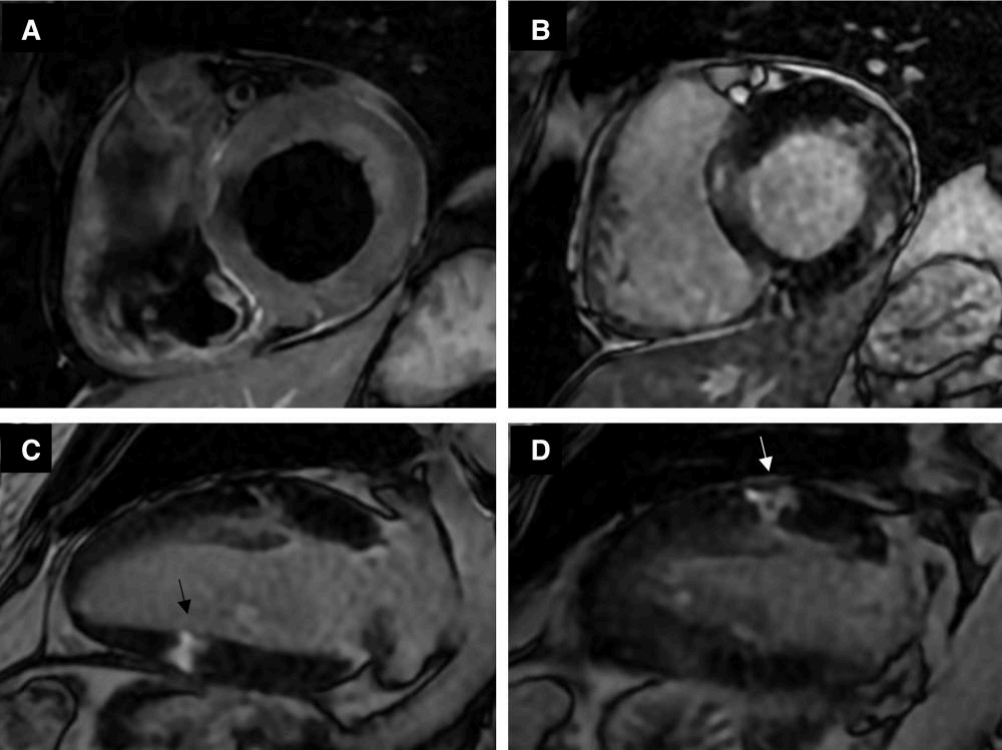
CMR, multifocal ischaemic changes. CMR T2-weighted image (*A*) and myocardial delayed enhancement images (*B–D*) showing ischaemic changes in the left anterior descending and circumflex artery territories.

Furthermore, on T2-weighted images, oedema was documented in the lateral basal and mid-ventricular wall and focal oedema in the inferior mid-ventricular wall. On delayed enhancement images, abnormal transmural enhancement was demonstrated in parts of the lateral mid-ventricular wall and focal enhancement in the inferior mid-ventricular wall. This was associated with mild diffuse subendocardial hyperintensity in the anterior mid-ventricular wall and transmural hyperintensity in the remaining anterolateral and inferior mid-ventricular walls. Cine-CMR images showed regional wall motion abnormalities in the left anterior descending and circumflex artery territories (*Figure 3* and *Video 5*). This multivessel ischaemic pattern was consistent with embolic myocardial infarction, so the diagnosis of embolic MINOCA was established.

## Outcome and follow-up

On Day 5, the patient underwent surgical removal of a gelatinous left atrial mass through a biatrial transseptal approach. The postoperative course in the cardiac surgical intensive care unit was uneventful. On postoperative Day 2, the patient was transferred to the cardiac surgery unit and then to a cardiac rehabilitation unit.

The diagnosis of cardiac myxoma was then confirmed by histopathological examination, which shows cellular elements with oval nuclei and finely dispersed chromatin, interspersed in a myxoid matrix.

On 6-month follow-up, the patient did not experience any cardiovascular adverse event or tumour recurrence at transthoracic echocardiographic evaluation.

## Discussion

MINOCA is a working diagnosis that applies in the presence of criteria for acute myocardial infarction (MI) and no coronary artery stenosis ≥50% on coronary angiography in the absence of alternative non-ischaemic causes for the acute presentation.^1^ MINOCA accounts for about 5–6% of all patients undergoing coronary angiography for MI, and a multimodal diagnostic approach is needed to tailor treatment based on pathophysiologic mechanisms, based on coronary functional assessment through acetylcholine (ACh) provocative testing,^2^ optical coherence tomography (OCT), and CMR.^3^ Causes of MINOCA include epicardial or microvascular coronary artery spasm, non-significant atherosclerotic plaque disruption rupture/erosion, non-obstructive coronary artery dissection, and coronary embolism.^1^

Cardiac myxomas are the most common primary cardiac tumours, accounting for 50–85% of benign cardiac neoplasms. They arise from mesenchymal cell precursors and form intracavitary masses, mainly in the left atrium (75% of cases), where they are often attached to the fossa ovalis by a stalk/peduncle. However, myxomas may also arise in the right atrium, left atrial free wall or appendage, and rarely in the ventricles or valve leaflets.^4,5^

The estimated prevalence of cardiac myxomas is 0.03%, with an annual incidence of 0.5–1 case per million. Myxomas can affect any age but are most commonly diagnosed in middle age and have a female preponderance (female-to-male ratio of approximately 3:1).^7^ About 7% of patients diagnosed with cardiac myxoma are affected by Carney complex, an autosomal dominant syndrome associated with endocrine hyperactivity and lentiginosis.^4^

Intracardiac obstruction may be the initial manifestation (mainly associated with polypoid tumours), with valvular obstruction often involving the mitral valve during diastole, which may lead to syncopal episodes, heart failure symptoms, and the development of progressive pulmonary hypertension.^4,5^

Myxomas may present as polypoid or papillary (or villous), the latter being particularly prone to give rise to embolic events due to their friable texture and tendency to fragment.^4,5^ Embolic events occur in 30–40% of myxoma patients and represent the ‘functional malignancy’ of myxoma.^4,5^ Myxoma-related coronary embolism is a rare phenomenon: in a cohort of 162 patients with cardiac myxoma, only one presented with coronary embolism (0.62%).^6^ This rarity is attributed to the small diameter of the coronary ostia, their right-angled junction within the aortic bulb, and the protection provided by the aortic valve cusps.^7^

Other reports of myocardial infarction likely due to embolism arising from cardiac myxomas have been previously described in the literature.^7^ Typically, CMR, when applied, has been used in such instances primarily to characterize the cardiac mass rather than to confirm an embolic ischaemic pattern in MINOCA.^8^ In our case, however, CMR also played a key role in supporting the diagnosis of coronary embolism as the underlying mechanism of MINOCA, revealing ischaemic involvement of multiple coronary territories.

On the other hand, ACh intracoronary testing and OCT were not performed in our case, as a vasomotor abnormality was not suspected based on the clinical presentation, nor was plaque instability indicated by the angiographic findings.

In conclusion, in this case report, we have documented a rare occurrence of MINOCA due to coronary embolism from a left atrial myxoma. The identification of an embolic pattern through CMR is considerably rare in such clinical scenarios, making its application in this case particularly valuable and underscoring its crucial role in the diagnostic process for MINOCA.^9^

## Data Availability

No new data were generated or analysed in support of this research.

## Lead author biography

Annalisa Pasquini, MD, PhD, is a consultant cardiologist at IRCCS Foundation Agostino Gemelli Hospital at the Catholic University of the Sacred Heart, Rome, Italy. Her clinical focus is on 2D/3D transoesophageal echocardiography and heart failure. She works with a high-level team whose main research activities and expertise focus on surgical and interventional cardiac imaging. She obtained her PhD at the Catholic University of the Sacred Heart, Italy 2017–2021. Speaker at Congresses and Transoesophageal Echocardiography Courses. She is a Member of the Working Group of Echocardiography and Group of Valvulopathies (from 2023), Italian Society of Cardiology (SIC); ESC Professional Member; EACVI Silver Member; and ESC HFA Member (ESC ID 753407).
